# “Now that the baby is out, I can be vaccinated”: a qualitative study on COVID-19 vaccine hesitancy in pregnant women in Kilifi, Kenya

**DOI:** 10.3389/fpubh.2026.1730282

**Published:** 2026-02-06

**Authors:** Angela Koech, Onesmus Wanje, Grace Mwashigadi, Geoffrey Katana, Richmond Mdindi, Peter Mwangome, Rachel Craik, Marianne Vidler, Mai-Lei Woo Kinshella, Peter von Dadelszen, Kirsty Le Doare, Marleen Temmerman, The periCOVID-Africa Consortium, Bridget Freyne, Kondwani Kawaza, Samantha Lissauer, Kalvor Sommerfelt, Melani Etti, Robert Mboizi, Stephen Cose, Victoria Nankabirwa, Lauren Hookham, Joseph Ouma, Gordon Rukondo, Madeleine Cochet, Merryn Voysey, Liberty Cantrell, The PRECISE Network, Patricia Okiro, Geoffrey Omuse, Shilla Dama, Nathan Barreh, Sharon Konde, Alice Kombo, Grace Maitha, Moses Mukhanya, Robin Okello, Juma Gumbo, Joseph Mutunga, Isaac Mwaniki, Marvin Ochieng, Emily Mwadime, Umberto D’Alessandro, Anna Roca, Hawanatu Jah, Andrew Prentice, Melisa Martinez-Alvarez, Brahima Diallo, Abdul Sesay, Sambou Suso, Yahaya Idris, Baboucarr Njie, Fatima Touray, Fatoumata Kongira, Modou F. S. Ndure, Gibril Gabbidon, Lawrence Gibba, Abdoulie Bah, Yorro Bah, Esperança Sevene, Corssino Tchavana, Salesio Macuacua, Anifa Vala, Helena Boene, Lazaro Quimice, Sonia Maculuve, Inacio Mandomando, Laura A. Magee, Marie-Laure Volvert, Hiten Mistry, Thomas Mendy, Donna Russell, Prestige Tatenda Makanga, Liberty Makacha, Reason Mlambo, Lucilla Poston, Rachel Tribe, Sophie Moore, Tatiana Salisbury, Aris Papageorghiou, Alison Noble, Hannah Blencowe, Veronique Filippi, Joy Lawn, Matt Silver, Joseph Akuze, Ursula Gazeley, Judith Cartwright, Guy Whitley, Sanjeev Krishna, Jing (Larry) Li, Jeff Bone, Domena Tu, Ash Sandhu, Kelly Pickerill, Carla Carillho, Benjamin Barratt, Amina Abubakar, Akbar K. Waljee

**Affiliations:** 1 Centre of Excellence for Women and Child Health – Aga Khan University, Nairobi, Kenya; 2 Department of Obstetrics and Gynecology, Aga Khan University, Nairobi, Kenya; 3 Kilifi County Department of Health and Sanitation Services, Kilifi, Kenya; 4 Department of Women and Children’s Health, Kings College London, London, United Kingdom; 5 Department of Obstetrics and Gynaecology, University of British Columbia, Vancouver, BC, Canada; 6 Pediatric Infectious Diseases Research Group and Vaccine Institute, St. George’s University of London, London, United Kingdom; 7 Faculty of Medicine and Health Sciences, Ghent University, Ghent, Belgium

**Keywords:** COVID-19, Kenya, pregnancy, vaccination, vaccine acceptance, vaccine hesitancy

## Abstract

COVID-19 vaccines are safe and effective in pregnancy, but vaccine hesitancy limits uptake and effectiveness. This study explored COVID-19 vaccine hesitancy in pregnancy in Kilifi, coastal Kenya, to elicit reasons for vaccine hesitancy and acceptance, and to compile misconceptions around vaccination in pregnancy. Twenty-three in-depth interviews were conducted with pregnant women, mothers who had given birth in the previous 2 years and health workers (community health promoters, nurses, and supervisors). Data were analyzed using thematic template analysis based on the Vaccine Hesitancy Determinants Matrix. Concern about vaccine safety for the unborn baby was a major driver of hesitancy. Many pregnant women had limited knowledge of the potential benefits to the unborn baby, leading to postponing vaccination until after pregnancy. The initial government exclusion of pregnant women from vaccination led many to believe that vaccines were unsafe in pregnancy, long after the eligibility was revised. Aggressive promotion of the vaccine by the government was a source of mistrust and misconceptions. Integrating COVID-19 vaccination into routine antenatal care improved acceptance and development and dissemination of local guidelines boosted healthcare workers’ confidence in offering vaccines to pregnant women. Future rollouts of vaccines for pregnant women should consider vaccination within antenatal care clinics alongside other routine pregnancy vaccines to enhance vaccine acceptance.

## 1 Introduction

COVID-19 was declared a pandemic by the World Health Organization in March 2020, with the first confirmed case in Kenya reported in March 2020 (1). Among pregnant women, rates of serious maternal morbidity, maternal mortality, stillbirth, pre-eclampsia and preterm birth are significantly higher among those diagnosed with COVID-19 compared to those without COVID-19 (2, 3). Vaccination remains a key strategy to sustainably control COVID-19 in the long term. There is growing evidence of the safety and effectiveness of COVID-19 vaccines during pregnancy (4, 5); however, substantial COVID-19 vaccine hesitancy among pregnant women (6, 7) limits uptake and effectiveness.

As pregnant women were excluded from the initial COVID-19 vaccine trials, the safety of the novel mRNA vaccines among pregnant women had not been proven by the start of vaccine roll-out (8). As a result, many initial vaccination programs did not include pregnant women. In Kenya, pregnant women were not targeted in the initial vaccine roll-out that began in March 2021 (9). The professional body of obstetricians and gynecologists in Kenya recommended vaccination in pregnant and breastfeeding women in August 2021 (10) and a similar directive from the Ministry of Health was issued in December 2021 (11).

Vaccine hesitancy is defined as uncertainty or refusal of the vaccine, despite availability of vaccine services (12). Vaccine hesitancy varies in different geographical contexts and different population groups (13, 14). Compared with the general population, pregnant women tend to have higher vaccine hesitancy for all / most vaccines that is driven primarily by concerns regarding maternal and perinatal safety (15). There are several studies on COVID-19 vaccine hesitancy in the general population and in pregnancy, but few have been conducted in Africa. This study focuses on this special group and seeks to understand the factors behind it in a specific geographical setting where disease exposure was high (16) but vaccination rates were low (17). Understanding the contextual factors would guide policy makers to design approaches to improve vaccine uptake among this vulnerable population. In addition, lessons learnt from COVID-19 vaccination would be applicable in future vaccine roll-outs for vaccines targeting pregnant women (18).

This study aimed to elicit the reasons for COVID-19 vaccine hesitancy and acceptance among pregnant women in Kilifi and to compile misconceptions regarding vaccination in pregnancy.

## 2 Materials and methods

### 2.1 Study design

This was a qualitative study with an interpretivist research approach. The phenomenon of interest was COVID-19 vaccine hesitancy and acceptance during pregnancy. We sought to obtain perspectives from both pregnant and recently pregnant women and health providers using in-depth interviews. This paper presents the qualitative findings from a mixed methods study on COVID-19 vaccine hesitancy. The project also included a quantitative cross-sectional survey to determine prevalence in the same setting, with this qualitative component exploring the deeper contextual factors and perspectives.

### 2.2 Study setting

**Figure 1:**
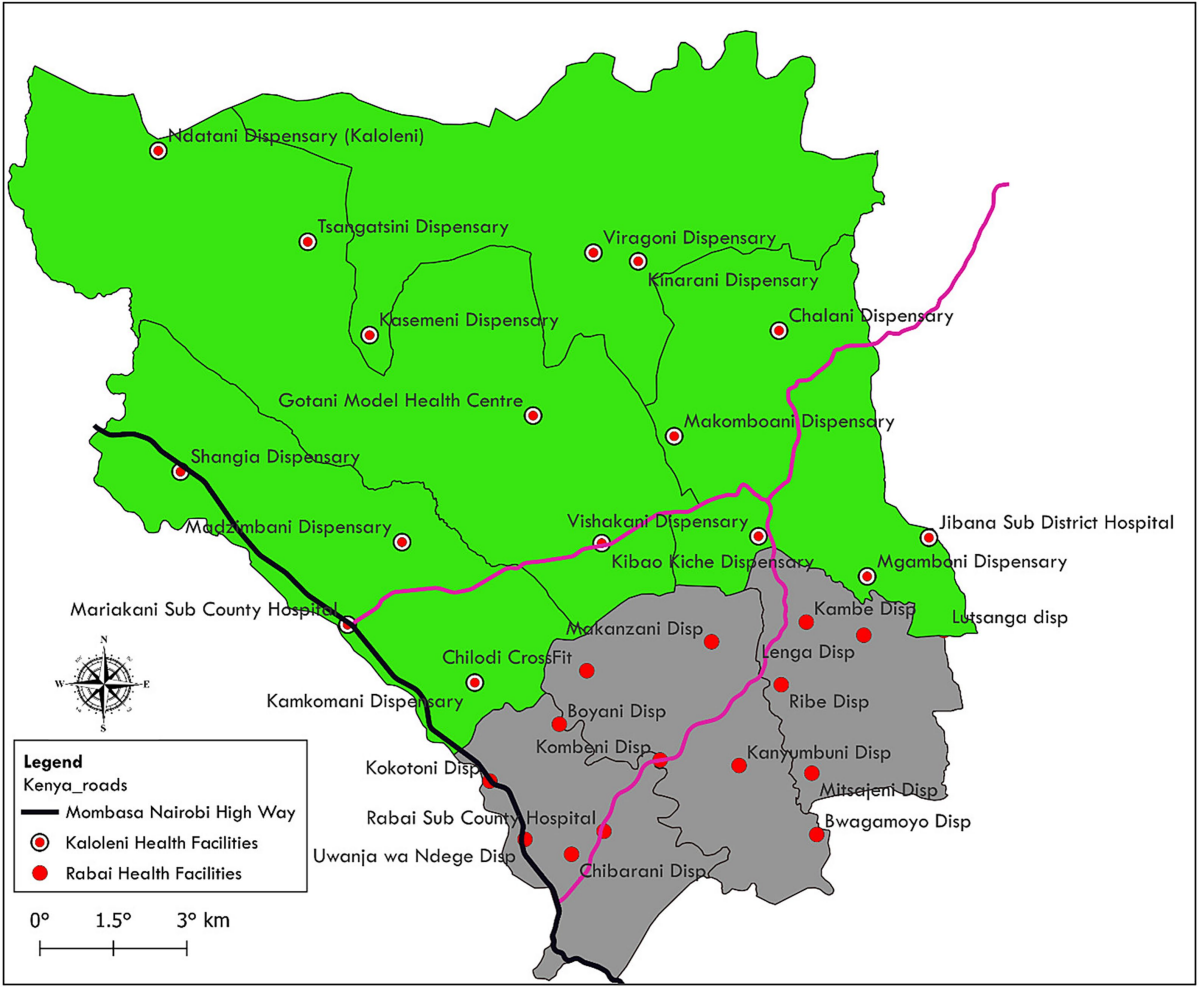
Map of Kaloleni and Rabai sub county showing health facilities.

The study was conducted in health facilities in Kaloleni and Rabai sub-counties in Kilifi County, Kenya. In this area, women of reproductive age make up 23% of the population and high antenatal clinic attendance—99% of pregnant women visit health facilities for antenatal care at least once (19). A total of 31 health facilities were selected for the mixed method study. A health facility was selected if it was a public facility managed by the Kilifi County Department of Health and if it was actively providing antenatal care during the study period. At the time of the study, there were 15 public facilities in Rabai sub-county and 16 in Kaloleni sub-county that met these criteria. Public health facilities are widely distributed in the two sub-counties, in contrast to private facilities that were more concentrated in urban areas. The distribution of public health facilities is shown in Figure 1.

### 2.3 Study participants

We included pregnant and recently pregnant women, frontline health workers involved in administering vaccines and/ or educating community members about vaccination (nurses and community health promoters (CHPs)), healthcare managers who directly supervised the frontline health workers, and healthcare managers who were responsible for COVID-19 control programs and/or reproductive health programs in the two sub-counties.

Eight pregnant women and two mothers who had given birth in the previous 2 years were invited to participate in the study by trained research assistants (RAs). All participants were over the age of 16. They were recruited during their routine outpatient antenatal care visits at the selected health facilities. RAs approached potential participants in the waiting areas of these facilities, explained the study’s purpose, and obtained informed consent prior to conducting interviews. Ethical approval was obtained from the Institutional Scientific and Ethics Research Committee of Aga Khan University.

To ensure a representative sample, purposive sampling was employed. This involved selecting health facilities to include a mix of urban and rural locations, as well as high- and low-volume facilities. The sampling strategy also aimed to achieve a wide range of age groups among participants and a balanced representation of the two sub-counties (with at least one-third of participants originating from each sub-county). Ten health facilities in the two sub counties (out of a possible 31) were represented in the qualitative study. Recruitment continued until data saturation was achieved, defined as the point where no substantively new themes related to COVID-19 vaccination decision-making were identified in subsequent interviews. The final sample size was determined by the richness of the individual cases (information power) rather than statistical power.

Thirteen healthcare workers were recruited at their respective workplaces by the RAs. These included four nurses, four community health promoters and five supervisors. Eligibility required that healthcare workers had been employed in either sub-county for a minimum of 1 year prior to recruitment.

### 2.4 Data collection

Recruitment and data collection took place between 05-12-2022 and 23-12-2022. At that time, COVID-19 vaccines were widely available in most public health facilities. In-depth interviews (IDIs) were conducted in private areas within health facility compounds. Trained interviewers conducted the IDIs using an IDI guide that was specific for each participant group (see Supplementary material S1) and using probes as necessary. Questions addressed the following key domains; participant profiles, knowledge and awareness, decision-making drivers, social/cultural influences, and information sources. For pregnant and recently pregnant women, interview questions included knowledge about COVID-19 and COVID-19 vaccination in pregnancy, willingness to accept the vaccine if offered, personal experiences around vaccination, beliefs regarding COVID-19 and COVID-19 vaccination in pregnancy and sources of information about COVID-19 vaccination. For health care workers, interview questions explored personal experiences regarding COVID-19 vaccination in pregnancy, procedures for counseling for and administering the COVID-19 vaccine in pregnancy, reasons why pregnant women accepted or declined COVID-19 vaccination, beliefs and misconceptions regarding the vaccine in the community, and sources of information about COVID-19 vaccination. IDIs were conducted primarily in Swahili for the pregnant and recently pregnant women and a mix of English and Swahili for health care workers. IDIs with pregnant and recently pregnant women were on average 28 min in length, while those with health care workers were on average 33 min in length. A meal and a travel reimbursement were provided after the session. IDIs were conducted by skilled researchers who were all fluent in the language of the interviews. Field notes were taken after each interview and reviewed during analysis to give context to the interviews.

### 2.5 Data management and analysis

All interviews were audio recorded, transcribed verbatim and translated to English where applicable. Names and personal identifiers were not transcribed. All transcripts were reviewed and compared with audio recordings to ensure accuracy of transcription and translation. We used NVivo 12 (QSR International now Lumivero, 2018) to manage the data. The analysis was a collaborative and iterative process. Initially, two researchers independently coded a sample of transcripts to develop a preliminary codebook. The team then met to discuss, compare and refine the codes, resolving discrepancies through consensus and establishing clear definitions. This codebook was used to code the remaining transcripts. Regular analysis meetings were held throughout the process to discuss the application of codes, group codes into broader themes, and collectively interpret the findings. Confidentiality and safe storage of the data were ensured by de-identifying transcripts and storing them in password-protected devices accessible only to members of the research team.

We used template analysis to analyze the interview data (20). Template analysis is a type of thematic analysis where researchers can use themes identified from previous work to develop an initial coding template. For this study, we used the Vaccine Hesitancy Determinants Matrix developed by the SAGE working group on vaccine hesitancy (12) (Supplementary material S2). This matrix groups factors influencing the behavioral decision to accept, delay or reject some or all vaccines under three categories: contextual influences, individual and group influences and vaccine/vaccination specific issues. The matrix has been used widely to describe findings of qualitative data on vaccine hesitancy for various vaccines and participant groups (21, 22). We applied the matrix separately for vaccine hesitancy and vaccine acceptance. We used the SAGE matrix as an initial deductive coding framework. However, to ensure local specifics were captured, inductive codes were developed for themes that did not fit neatly within the framework. These were initially coded as free-standing nodes and later integrated into the final template through discussion and consensus. (AK, OW, GM, GK, RM, PM and MWK). Throughout the analysis, we triangulated views from the different participant groups highlighting any differences and similarities in the findings. Excerpts from the transcripts were selected to illustrate the various themes and sub-themes.

### 2.6 Researcher characteristics and reflexivity

All data collectors were Kenyan and familiar with both the Kenyan health care system and study area. All, except one, had had lengthy work experience conducting maternal health research in health facilities and field sites in the study area both before and during the COVID-19 pandemic. All, except one, of the IDIs with pregnant and recently pregnant women were conducted by female interviewers. We believe that this improved women’s comfort with expressing their views. All, except one, of the IDIs with health care workers were conducted by persons with no clinical background. We believe that this encouraged participants to respond to questions without fear of judgment of the accuracy of their clinical knowledge. Researchers GK, RM and PM, had direct supervisory roles with the health workers who participated in the study and, therefore, did not conduct the interviews, but rather participated in the data analysis. Specific job roles and respective health facilities for the health workers were not asked about during the interviews and were not transcribed to assure health workers of their confidentiality and remove any fear of victimization.

The Standards for Reporting Qualitative Research (SRQR) Checklist was used to guide the reporting (Supplementary material S3).

## 3 Results

### 3.1 Participants characteristics

A total of 23 IDIs were conducted; eight with pregnant women, two with recently pregnant women and 13 with health care workers. All women enrolled were married or cohabiting, including the teenage mothers (2), which is representative of the study area. Five women were housewives, while the other five engaged in small businesses. Detailed participant characteristics are given in Table 1.

**Table 1:** Participants characteristics for in-depth interviews.

Characteristic	Pregnant and recently pregnant women (6–12 months) *N* = 10	Community health promoters *N* = 4	Nurses *N* = 4	Supervisors *N* = 5
Age (Years)				
16–19	2			
20–29	4			
30–39	4			
Marital status				
Married or cohabiting	10			
Not married	0			
Number of children				
0	1			
1–5	8			
6–10	1			
Occupation				
Housewife	5			
Small business (self-employed)	5			
Pregnancy Status				
Pregnant	8			
Recently pregnant	2			
Vaccination status				
Vaccinated	4			
Not vaccinated	6			
Sub county of residence (for women) and sub county of workplace (health workers)				
Kaloleni sub county	5	2	2	3
Rabai sub county	5	2	2	2
Nature of locality of the health facility of enrolment (for women) or workplace (health workers)				
Urban	1	0	1	
Peri-urban	2	1	1	
Rural	7	3	2	
Years worked in the study area				
0–5 years		1	2	1
5–10 years		1	1	1
More than 10 years		2	1	3

The grey color indicates categories that are not applicable to that specific participant group.

### 3.2 COVID-19 vaccine hesitancy

Despite wide vaccine availability in the study area, the majority of pregnant women interviewed stated that they were unwilling to receive the COVID-19 vaccine. Health workers also reported this.

“*The mothers still have a little bit of fear for the vaccine. Yeah, so the uptake of covid-19 vaccine to the pregnant mothers is still low. Yeah.” Frontline nurse 4.*

Table 2 summarizes the reasons for hesitancy.

**Table 2:** Reasons for COVID-19 vaccine hesitancy.

Reasons for COVID-19 vaccine hesitancy among pregnant women	
Contextual influences	Cultural context: Husbands as primary decision makers declining vaccination for their wives Religion and faith Pre-existing mistrust in the government
Individual and group influences	Pregnancy itself ◦ Pregnant women’s concerns regarding the safety of the vaccine to themselves ◦ Pregnant women’s concerns regarding the safety of the vaccine to their unborn baby Inaccurate knowledge on eligibility to receive the vaccine Low risk perception of COVID-19 and belief that COVID-19 does not exist Mistrust brought about by the government’s promotion of the vaccine Rumors and spread of misconceptions about the vaccine Lack of decision-making autonomy ◦ Husbands prohibiting vaccination for their wives ◦ Family members and others prohibiting or discouraging vaccination Contradicting opinions or disagreements about the vaccine Anti-vaccination messages from religious leaders
Vaccine/vaccination-specific issues	Exclusion of pregnant women in initial vaccine roll-out. Lack of clear guidelines about vaccination of pregnant women Insufficient information given on the safety of the vaccine or reasons for vaccination

We explored the factors influencing vaccine hesitancy and organized themes according to the three determinants of the SAGE model which are the contextual influences, individual and group influences, and vaccine/vaccination-specific issues.

#### 3.2.1 Contextual influences of vaccine hesitancy

A key contextual factor that led to vaccine hesitancy in the study area was the common cultural practice whereby women deferred decision making to their husbands, including decisions on healthcare seeking. Pregnant and recently pregnant women and healthcare workers reported scenarios when women postponed vaccination to obtain consent from their husbands or when husbands prohibited their wives from receiving the vaccine.

*“My husband was saying I should not do anything without consulting him. You know I cannot make such a decision alone. They (the vaccines) may affect you or they may not; and if they affect you negatively then he will say that he warned me against it.” Pregnant woman 3.*

Pre-existing mistrust in the government was a significant driver of vaccine hesitancy. This mistrust fueled the misconception that the vaccine was a covert government plan to control population growth through contraceptives or by causing infertility. Ironically, the government’s strong promotion of the vaccine intensified these suspicions of a hidden agenda. This specific fear was widely reported by both pregnant women and healthcare workers.

*“Others say it is a family planning method and if you get it, you will no longer give birth. Others even say it is to reduce the population so if you get the vaccine, you will not live for the years you were meant to live and you will die early.” Community health promoter 2.*

*“It is said that the government brought the vaccine to reduce the population, so everyone is afraid” Pregnant woman 5.*

The misconception that COVID-19 did not exist was also a driver for the hesitancy. The idea that the pandemic was a hoax is a narrative that was created and sustained by certain media spaces and word-of-mouth.

*“What I have heard about it especially in the community perspective is that there is no COVID at all.” Supervisor 1.*

*“The elders in my community and that of my husband are celebrating because in this area where we live has not had even a single case of COVID. We only see it in the TV, so we think that the people were just paid to lie to us who do not understand.” Pregnant woman 5.*

#### 3.2.2 Individual influences of vaccine hesitancy

The primary influence at the individual level was the state of pregnancy itself. Many pregnant women were concerned for their own safety and that of their unborn babies. They described concerns that a vaccine which caused side effects in non-pregnant women would probably cause more severe side effects or consequences for pregnant women. The vaccine was commonly described as ‘too strong’, and women had fears of harmful outcomes for their babies such as premature birth, miscarriage and fetal malformations.

*“I thought, if I got vaccinated, I might lose my baby. Because I am told it (the vaccine) is strong. That is what was worrying me.” Recently pregnant woman 1.*

*“Because I have carried something delicate in my womb.” Recently pregnant woman 1.*

Both recently pregnant women interviewed were not vaccinated and had declined vaccination during pregnancy. However, both stated that they were willing to be vaccinated in their post-pregnancy state.

*“What contributed (to me changing my mind) was that I saw I was no longer pregnant. There was nothing inside me that it (the vaccine) could harm. I was defending the creature (unborn baby), because I thought it could be said to be good but then have side effects on the baby. But now that the baby is out, I can be vaccinated.” Recently pregnant woman 1.*

In addition, both perceptions of COVID-19 as a disease and the perceived risk of contracting it, influenced vaccine hesitancy. Earlier in the pandemic, many community members in the study settings (especially in the rural areas) felt that COVID-19 did not exist because they had not seen any cases. Some pregnant women did not see the need to receive the COVID-19 vaccine because of this perceived low risk of disease. In addition, a few respondents described a shift toward declining vaccine acceptance due to the decreasing cases of COVID-19 disease and reduced emphasis on public health control measures. Risk of disease was perceived more for the mother and less for her unborn baby. Only one woman reported benefits of the vaccine to the unborn baby.

*“Others do not see the importance because they say ‘I have not seen anyone in this area contract COVID so we do not know how it looks like. Hence whether I get vaccinated or not I will not get COVID since it’s not there” Community health promoter 2.*

*“…when we started there were those things like sanctions that were put. …now people relaxed because they are like ‘COVID is not there anymore’, so even that push that was there is no longer there.” Frontline nurse 4.*

#### 3.2.3 Group influences of vaccine hesitancy

At the group level, women’s decisions were often influenced by advice from their family members and others. They described hearing misconceptions and rumors spread about alleged harmful effects of the vaccine and that these led them not to receive the vaccine. Some vaccinated persons would talk about their experience with vaccination and its side effects and instill fear in others who had not yet received the vaccine.

*“I registered my name (for vaccination). On the day I was to go (for vaccination) another woman told me that she was vaccinated, and the vaccine did this and that. So, I became afraid and decide that I would not go.” Recently pregnant woman 2.*

*“We were told to get the injection and they even came to the school. When I told my mother she said I should not get the injection. … She said the injections could be poisonous.” Pregnant woman 6.*

*“People had the fear why even the government was forcing them (to take the vaccine)” Supervisor 5.*

The perceived risks and fears held by individuals were also key drivers for hesitancy. Many of the misconceptions were exaggerations of known side effects of the vaccines. Misconceptions that the COVID-19 vaccine caused adverse birth outcomes and pregnancy loss was commonly described. Others on harmful effects of the vaccine on the vaccine recipient—e.g. causing blood clots and that the COVID-19 vaccine causes deformities in the baby

*“It is said that when you are injected with that vaccine, especially when you are pregnant the vaccines are very strong and so if you are not as strong and plus having a baby in the belly that is not fully developed then you can have a premature birth. Or still you can miscarry.” Pregnant woman 1.*

*“Others were like, they were injected with the COVID -19 vaccine and immediately blood started coming out from the body openings, you see. It was said that the injection went and cut down all the blood veins in the body.” Community health promoter 1.*

*“They say you can give birth to a deformed baby as the vaccines are not good. ‘Why should I give birth to a deformed child?’” Community health promoter 3.*

*“There is a mother who told me she does not want neither will she get the vaccine because she heard the vaccine is not good. When one gets the vaccine they will have a clot in their brain.” Nurse 1.*

*“First there was this rumour that if you get the injection then it can lead to severe illness, body ache, you may not be able to work with your hand and you may even die even after going to the hospital.” Community health promoter 3.*

The presence of contradicting opinions led to delays in deciding to receive the vaccine. Some of these conflicting messages were from family members, community members and religious leaders.

*“Because there are some that tell you ‘get vaccinated’ and others tell you ‘do not get vaccinated’. Recently pregnant woman 1.*

*“There is a pastor who says, ‘eee is there even a disease called Corona? (COVID-19)? There is no COVID disease. It is just (the president) wanting to make money, but there is no COVID. So the vaccine, isn’t necessary.’ Others (other pastors) say the vaccine is necessary because it is a disease.” Recently pregnant woman 2.*

Similarly, contradicting opinions were described by healthcare workers. There were some healthcare workers who themselves were vaccine hesitant and subsequently, hesitated to recommend or declined to administer the vaccines to pregnant women.

*“Of course it brought about disagreements, there was a divide among health care workers as others embraced it and got vaccinated … So, you get the one administering the vaccine has not been vaccinated and he says he will administer the vaccine, but he (himself/herself) will not get vaccinated. Even today there are health workers who have not been vaccinated.” Supervisor 4.*

*“They (pregnant women) will say they are not sure about the vaccine. It has not been well researched on people who are pregnant, so they still think it doesn’t make sense to them.” Supervisor 3.*

#### 3.2.4 Vaccine and vaccine-specific issues for hesitancy

Various aspects related to the vaccine, the delivery of information about the vaccine and the vaccination processes contributed to vaccine hesitancy. This was despite having contact with healthcare workers at both facility and community level. All women had heard about COVID and the COVID vaccine; however, in many cases the knowledge was shallow and lacked detail. Many did not know that pregnant women were eligible for vaccination. Women expressed the desire to know more so that they could make an informed decision.

*“I declined because I had not yet been taught, and I did not have sufficient awareness. I was just told, ‘Corona exists. One should be vaccinated and if you are not vaccinated you will get this and that. …’ If only there had been teachings that would help people to understand more.” Recently pregnant woman 1.*

*“They say that the vaccine is not harmful, but they should have given more explanation about the vaccine. But now, they don’t tell us why, just that the government has said you should be vaccinated.” Recently pregnant woman 1.*

Another important aspect was the nature of introduction of the COVID-19 vaccines. Exclusion of pregnant women from initial COVID-19 vaccination rollouts led to widespread confusion and uncertainty among both pregnant women and healthcare workers. The uncertainty decreased when new guidelines that recommended pregnant mothers to be vaccinated were developed and disseminated. However, it was evident in this study that some pregnant mothers still held onto the initial guidelines and believed that they were ineligible to receive the vaccine.

*“What stops them is what they were told before that pregnant women should not get the vaccine and so they have stuck with that. If you tell her to go get the vaccine, it is good, she says that (pregnant) women are not supposed to take it.” Community health promoter 3.*

A lack of clear communication created significant misunderstanding about the public health recommendations for COVID-19 vaccination in pregnant women. This issue aligns with the SAGE model’s determinant on the “strength of the recommendation and/or knowledge base of healthcare professionals,” as some women were given incorrect information regarding their eligibility to receive the vaccine.

*“I don’t understand but I heard that when one is pregnant they are not supposed to be injected with the vaccine, she might have a premature birth.” Pregnant woman 4.*

Misinformation about the vaccine’s technology was a key issue that challenged its scientific risk/benefit profile. A prominent example was the rumor that the COVID-19 vaccine could alter a person’s DNA

*“Someone else told me that it changes someone's DNA, and you see when your DNA is changed that means you are not the same person. … when you are vaccinated then you change to a monkey.” Supervisor 4.*

### 3.3 COVID-19 vaccine acceptance

While the predominant view was that of vaccine hesitancy, there were descriptions from some participants of vaccine acceptance and positive perceptions toward the vaccine. The factors that contributed to vaccine acceptance are summarized in Table 3.

**Table 3:** Reasons for COVID-19 vaccine acceptance.

Reasons for acceptance of COVID-19 vaccine among pregnant women	
Individual and Group influences	Safety of the vaccine for mother and unborn baby Perceived protection for mother and unborn baby Accurate knowledge regarding the vaccine and vaccination eligibility Observed high transmission and devastating effects of COVID-19 in the community Close contact with persons with COVID-19 disease Observing persons who have been vaccinated and not harmed by the vaccine Encouragement and support from family members, including spouses Pro-vaccination messages from religious leaders
Vaccine/vaccination-specific issues	Safety of the vaccine in pregnancy Increasing availability of vaccines at more health facilities Community sensitization by healthcare workers, especially community health promoters Development of guidelines regarding vaccination of pregnant women Integration of COVID-19 vaccination into routine vaccination services Counseling for and administering COVID-19 vaccines during routine antenatal care visits

#### 3.3.1 Individual and group influences for vaccine acceptance

Some pregnant and recently pregnant women were able to describe their decision-making process and how they made their own decisions about whether or not to be vaccinated. Key individual motivations for pregnant women being vaccinated were protection against COVID-19 disease and the vaccine’s safety for both the woman and her unborn baby. For others, it was fear of the disease and its consequences.

*“I will accept…First your health will be good and you will not be affected. Second, even if you are pregnant it will not hurt the baby, but it will also protect the baby and even if you have the virus in the body the baby will not be affected.” Pregnant Woman 5.*

*“Death is not good, my parents died (when I was young). So, when you’re told there is an illness it is better to go to hospital to protect yourself. Death is not good.” Pregnant woman 8 (Vaccinated, received booster dose while pregnant).*

Group influences from family members, other pregnant and non-pregnant women, and health workers were evident. Two women reported support from their spouses toward vaccination as a contributor for vaccination. These were through either direct advice from others or by making their own observations of the experiences of others. Close contact with, and hearing reports of, others who had contracted COVID-19 disease led them to understand the magnitude of risk. The rapid spread of the virus and the devastation it caused brought fear and drove vaccine acceptance. Similarly, observing others who had been vaccinated reassured them regarding the safety of vaccination.

*“Even my husband agreed and he said, ‘go and get the vaccine’.” Pregnant woman 5.*

*“...my aunt suffered from COVID -19 as I have just told you. And so, I saw how she was suffering. I feared and so I just wanted to protect myself.” Pregnant woman 1.*

*“I heard on the radio that corona is causing many deaths and now there is a medicine which is a vaccine that we should get. When the lady (community health promoter) came to tell me about it I had already heard on the radio, so I took my family and we received the vaccine. Pregnant woman 8.*

#### 3.3.2 Vaccine and vaccine-specific issue for acceptance

Various factors related to the design and delivery of the vaccination program were felt to have contributed to increasing vaccine acceptance. There was increased sensitization and education of the general population and pregnant women on the value of COVID-19 vaccination. This provided clarity on earlier concerns such as side effects in pregnancy. A distinct shift toward increasing vaccine acceptance over time was described by several participants, especially healthcare workers and supervisors.

*“The main thing that brought about the change is trying to give the knowledge to the mothers. Trying to educate them, trying to sensitize them about COVID-19 and the vaccine. The more knowledge you give them the more numbers you get at the facility.” Frontline Nurse 2.*

*“At least people have changed and during that time you had to convince people but now they come on their own and ask about the vaccine … This is a sign that the community has understood, and they have improved.” Supervisor 4.*

Some changes in the approach of the healthcare system toward delivery of the vaccines were said to drive increasing vaccine acceptance. Initially, vaccines were available only in larger health facilities but later they were distributed to all public facilities and more health workers given rights to administer the vaccines. Later, there was a decision to integrate COVID-19 vaccination into routine vaccination services and to deliver vaccines for pregnant women as part of routine antenatal care. With the integration, the COVID-19 vaccine was offered and discussed with all pregnant women at the antenatal clinic and administered alongside other pregnancy vaccines such as the tetanus vaccine. Healthcare workers and supervisors viewed this approach very positively.

*“Instead of going to look for the people in their houses or the meetings they come for, why not integrate them? ... So, we have decided to integrate the vaccine with the other vaccines and that is what makes (subcounty X) leading. …. we shared this idea and it seemed as a unique idea or way of improving vaccination.” Supervisor 4.*

*“… we need to capture the women and it’s always very difficult to go to the community and isolate pregnant women because pregnant women if you try to bring them together you will create a lot of rumours. ... So, the best thing is to strengthen the antenatal clinics and empower those who are doing the clinics with skills on how they can do mobilization, advocacy and behavior change for those who attended the clinics. And then use the same to be role models to others.” Supervisor 3.*

The important role played by practice guidelines was emphasized repeatedly. Official guidelines regarding vaccination from the Kenya Ministry of Health were a trusted source of information for healthcare workers. Healthcare workers frequently described the use of, and dependence on, the guidelines when communicating about and administering vaccines. In the absence of guidelines, there was confusion; however, following their introduction healthcare workers felt more confident to administer and communicate about vaccines.

*“Initially it was because some sections were saying it was not safe and some were saying it was. Until when there was a circular communicated from the ministry of health to confirm that pregnant women are to be given the vaccine. That is when it was cleared.” Frontline nurse 3.*

*“I would say the reason was, at first health care workers did not have the exact knowledge on how to guide these mothers. So, after the induction and maybe after the training and after the new research came up that they were saying that the vaccines are safe; so we have the knowledge and it becomes easy for us sensitize the mothers, to health educate them and we get the positive response from them.” Frontline nurse 4.*

## 4 Discussion

Our study demonstrates the central role of pregnant women’s individual concerns regarding vaccine safety, both to themselves and to their unborn baby, alongside group influences from families and other community members, in affecting both acceptance of and hesitancy regarding COVID-19 vaccination in pregnancy. Issues related to the roll out of the vaccine led to long term effects on vaccine hesitancy, while specific changes in the health system’s approach to vaccination improved vaccine acceptance over time.

Vaccine hesitancy was commonly described to arise from pregnant women’s concern regarding possible harm to their unborn babies from the vaccination. Myths and misconceptions revolved around exaggerated vaccine side effects and harmful consequences for pregnancy outcomes, further fueling hesitancy. COVID-19 vaccines had been publicized as beneficial to mothers with little messaging about benefits to babies and many women were unaware of the benefits of the vaccine to their unborn babies. Pregnant women opted to postpone vaccination until after delivery, feeling that they were preventing possible harm to the unborn baby, even if at the expense of the benefits of the vaccine to themselves. Safety concerns are a major factor affecting vaccine hesitancy in pregnancy in various settings in Kenya, Africa and beyond (22–26). In addition, studies comparing vaccine hesitancy in pregnant women vs. non-pregnant women have demonstrated higher hesitancy in pregnant women (27). This is not unique to the COVID vaccine and has been demonstrated for other vaccines administered in pregnancy (25). Practitioners and governments planning to introduce and promote vaccines in pregnancy should prioritize demonstrating both safety and benefits to the unborn baby. Several existing and new vaccines are administered to pregnant women in pregnancy primarily for the benefit of the baby. Acceptance of existing vaccines in pregnancy, such as the tetanus vaccine, is high in Kenya (28, 29). The aspect of ‘benefit to the child’ is a key feature of vaccines against Group B Streptococcus and respiratory syncytial virus that are yet to be introduced in the study area. Therefore, appropriate delivery of this message to pregnant women could promote higher vaccine acceptance.

Similar to other studies (22, 24), our study described various myths and misconceptions about the vaccine and their influence they have on vaccine hesitancy. Some misconceptions were exaggerations or distortions of known side effects of the vaccines. Healthcare workers and other educators should be cautious when delivering messages about known side effects and should address misconceptions specific to their contexts directly.

From a health systems perspective, the initial exclusion of pregnant women from vaccination programs led to a lasting effect on vaccine hesitancy. This exclusion was understood to mean that the vaccines were not safe for pregnant women rather than that there was limited information on safety at that time. This illustrates how structural barriers specifically the initial exclusion policies can create a vacuum of trust that is difficult to reverse, even after guidelines are updated. Similar to other studies (30), pregnant women maintained the initial understanding of ineligibility for vaccination despite a change in the guidelines. Vaccine developers and researchers can prevent this barrier to vaccination by including pregnant women in vaccine trials as early as possible and ideally before roll-out (31).

Despite the high vaccine hesitancy, a remarkable shift toward increasing vaccine acceptance was described in this study. One of the drivers was the positive community influence from others who had received the vaccine without suffering harm. This community influence, described in other studies (24, 26, 30), can be leveraged by enlisting vaccinated pregnant women as champions for vaccination in their communities.

The role of development and dissemination of clear guidelines on vaccination of pregnant women cannot be overstated. Guidelines increased the confidence of healthcare workers in administering the vaccine and communicating about the vaccine and probably improved vaccine acceptance. A study that was conducted in Kenya in December 2021, shortly after the Ministry of Health issued a directive encouraging vaccination of pregnant women, showed that many health workers found uncertainty over eligibility of pregnant women for vaccination and health worker hesitancy in administering vaccines to this group (30). In contrast, all health workers interviewed in our study were clear about the eligibility of pregnant women for COVID-19 vaccination and many delivered information about the vaccine confidently. This reliance on guidelines was particularly evident among nurses, who used them to establish clinical authority. In contrast, CHPs operated outside the clinical setting and faced the direct challenge of debunking community-specific rumors, often without the immediate support of official documentation.

Integration of COVID-19 vaccination in routine antenatal care was one of the factors thought to drive increasing vaccine acceptance in pregnant women. In Africa, maternal vaccine acceptance of other vaccines is high (29, 32). In Kenya, as many as 75% of pregnant women had received sufficient tetanus toxoid vaccine in their most recent pregnancy (19). Delivering COVID vaccines alongside other routine vaccines could reduce anxiety over the safety of vaccination and reduce misconceptions brought about by aggressive vaccine campaigns. Earlier integration with routine vaccination should be considered in future introduction of vaccines that include pregnant women.

### 4.1 Strengths of this study

This study was conducted at a time when vaccines were widely available in all public facilities, including both urban and rural areas, making it an appropriate time to investigate vaccine hesitancy. Guidance in favor of vaccination of pregnant women had been developed and disseminated widely. This study provides a window into a period in the pandemic when other public health measures against COVID had declined but vaccination remained important particularly for high-risk groups such as pregnant women. We collected data in several public facilities in the two sub-counties and in both urban and rural settings; therefore, our data are representative of the diverse settings in this region.

### 4.2 Limitations of this study

Data collection was conducted at health facilities, and this may have influenced participant responses in favor of vaccination. We attempted to mitigate this bias by reassuring participants of confidentiality and not collecting any personal identifiers. This recruitment strategy, while allowing us to reach a significant number of pregnant women and gather detailed information on their attitudes and experiences, introduces a potential selection bias. As such, the perspectives of women with limited access to healthcare, or those who actively avoid such facilities, may be underrepresented in our sample. This is a critical consideration, as these marginalized groups may experience unique barriers to vaccination and exhibit higher levels of vaccine hesitancy.

### 4.3 Implications for policy and practice

Findings of this study give us an opportunity to make recommendations on approaches for future vaccine introductions to reduce vaccine hesitancy in pregnancy. Based directly on our participants’ accounts, we recommend that messaging around vaccination in pregnancy includes information on both maternal and fetal benefits. Detailed safety information should be provided, and specific myths and misconceptions addressed directly. Furthermore, vaccination guidelines must be developed and circulated widely to all health workers before rollout to ensure confidence.

Broader recommendations supported by the wider literature suggest that vaccines for pregnant women should be introduced as part of routine antenatal care where trust is already established rather than through aggressive vaccination campaigns outside of health facilities. Additionally, vaccines should be tested early in pregnant women to prevent the lag in eligibility that fuels hesitancy.

## Data Availability

The original contributions presented in the study are included in the article/Supplementary material, further inquiries can be directed to the corresponding author.

## Group members of the periCOVID-Africa consortium

Bridget Freyne, Kondwani Kawaza, Samantha Lissauer, Kalvor Sommerfelt, Melani Etti, Robert Mboizi, Stephen Cose, Victoria Nankabirwa, Lauren Hookham, Joseph Ouma, Gordon Rukondo, Madeleine Cochet, Merryn Voysey, and Liberty Cantrell.

## Group members of the PRECISE Network consortium

Patricia Okiro, Geoffrey Omuse, Shilla Dama, Nathan Barreh, Sharon Konde, Alice Kombo, Grace Maitha, Moses Mukhanya, Robin Okello, Juma Gumbo, Joseph Mutunga, Isaac Mwaniki, Marvin Ochieng, Emily Mwadime, Umberto D’Alessandro, Anna Roca, Hawanatu Jah, Andrew Prentice, Melisa Martinez-Alvarez, Brahima Diallo, Abdul Sesay, Sambou Suso, Yahaya Idris, Baboucarr Njie, Fatima Touray, Fatoumata Kongira, Modou F. S. Ndure, Gibril Gabbidon, Lawrence Gibba, Abdoulie Bah, Yorro Bah, Esperança Sevene, Corssino Tchavana, Salesio Macuacua, Anifa Vala, Helena Boene, Lazaro Quimice, Sonia Maculuve, Inacio Mandomando, Laura A. Magee, Marie-Laure Volvert, Hiten Mistry, Thomas Mendy, Donna Russell, Prestige Tatenda Makanga, Liberty Makacha, Reason Mlambo, Lucilla Poston, Rachel Tribe, Sophie Moore, Tatiana Salisbury, Aris Papageorghiou, Alison Noble, Hannah Blencowe, Veronique Filippi, Joy Lawn, Matt Silver, Joseph Akuze, Ursula Gazeley, Judith Cartwright, Guy Whitley, Sanjeev Krishna, Jing (Larry) Li, Jeff Bone, Domena Tu, Ash Sandhu, Kelly Pickerill, Carla Carillho, Benjamin Barratt, Amina Abubakar, Akbar K. Waljee.
