# Supplementary material for: “Now that the baby is out, I can be vaccinated”: a qualitative study on COVID-19 vaccine hesitancy in pregnant women in Kilifi, Kenya
Source: Front Public Health. 2026 Feb 6;14:1730282. doi: 10.3389/fpubh.2026.1730282 (PMC12920558; doi:10.3389/fpubh.2026.1730282)
Supplement: Supplementary file 1 [file Data_Sheet_1.DOCX]

**“Now that the baby is out, I can be vaccinated”: A qualitative study on COVID-19 vaccine hesitancy in pregnant women in Kilifi, Kenya.**

**Supplementary material S1 - Interview guides**

**Interview guide for frontline health care workers (nurses and community health volunteers)**

1. Can you tell me a little about yourself and your role here at the health facility? How long have you been working at this health facility? In your current position?
2. What do you know about COVID-19 in pregnancy?
3. What do you know about the COVID-19 vaccine for pregnant women?
4. What is your experience counselling pregnant women on COVID-19 vaccines? What do you usually say?
5. What is your experience giving COVID-19 vaccines to pregnant women? As an estimate, how many COVID-19 vaccines have you provided to pregnant women?
6. If there was a pregnant woman at ANC who was interested in getting a COVID-19 vaccine, please tell me what would happen - walk me through the process or the various steps.
7. In your opinion, what prevents pregnant women from getting a COVID-19 vaccine?
8. In your opinion, what delays pregnant women from getting a COVID-19 vaccine?
9. In your opinion, what helps pregnant women to get a COVID-19 vaccine?
10. How do you feel when you help a pregnant women get a COVID-19 vaccine?
11. When did your facility start giving COVID-19 vaccines (month-year)?
12. How did pregnant women and their families react to getting a COVID-19 vaccine in the past when COVID-19 vaccines were first recommended for pregnant women?
13. What do pregnant women and their families say now about COVID-19 vaccines during pregnancy? If there has been a change, why do you think so?
14. How frequently do pregnant women accept the COVID-19 vaccine? For example, if there were five pregnant women, how many do you think would accept the COVID-19 vaccine?
15. In your experience, what are some of the reasons why pregnant women accept the COVID-19 vaccine?
16. Can you share a story that you experienced or heard happen at your health facility about a pregnant woman and/or her family who were really happy about getting a COVID-19 vaccine? Why were they so happy?
17. In your experience, what are some of the reasons why pregnant women refuse the COVID-19 vaccine?
18. Can you share a story that you experienced or heard happen at your health facility about a pregnant woman and/or her family who were upset or angry when counselled to get a COVID-19 vaccine? Why were they so upset?
19. Are there disagreements within families about pregnant women getting the COVID-19 vaccine? What happens when there are disagreements?
20. Are there disagreements between health workers about pregnant women getting the COVID-19 vaccine? Can you share any examples?
21. What beliefs about the COVID-19 vaccine during pregnant can be found in the community? Are there any beliefs that enable pregnant women to get the COVID-19 vaccine? Any beliefs or misconceptions that reduce COVID-19 vaccine uptake among pregnant women?
22. Where are pregnant women and their families getting information about the COVID-19 vaccine during pregnancy from? Where are people hearing misconceptions from?
23. Thank you for your time. Is there anything else you would like to share about the COVID-19 vaccine in pregnancy, or COVID-19 during pregnancy in general?

**Interview guide for pregnant and recently pregnant women**

1. Can you tell me a little about yourself, like where you are from and what you do for a living?
2. Do you have any children? How many? How old are they?
3. Are you married? What does your spouse do for a living?
4. Can you tell me what you know about COVID-19 during pregnancy? Where did you learn this information from? How did learning about COVID-19 in pregnancy make you feel?
5. Have you heard about getting the COVID-19 vaccine during pregnancy? Where did you hear about it from? What information was given to you about the COVID-19 vaccine during pregnancy?
6. Did the nurse at the ANC clinic talk to you about the COVID-19 vaccine? What did they say?
7. What were some of your first thoughts when you learned about the COVID-19 vaccine? What do you think of the COVID-19 vaccine now? If the perspectives changed: What led to your change in thinking about the COVID-19 vaccine?
8. Have you received the COVID-19 vaccine? Why or why not? If yes - were you pregnant when you received the vaccine?
9. If you were offered a COVID-19 vaccine during the current/most recent pregnancy, would you accept? Why or why not?
10. What do other pregnant women in your community say about getting the COVID-19 vaccine during pregnancy?
11. What does your family say about pregnant women getting the COVID-19 vaccine?
12. What do elders in your community say about pregnant women getting the COVID-19 vaccine?
13. What do religious leaders in your community say about pregnant women getting the COVID-19 vaccine?
14. What do traditional birth attendants in your community say about pregnant women getting the COVID-19 vaccine?
15. Are there disagreements in your community about pregnant women getting the COVID-19 vaccine? If there are disagreements, who do you believe to be telling the truth and why?
16. If you or people in your community said yes to pregnant women getting the COVID-19 vaccine, what would be some reasons why they accepted?
17. If you or people in your community said no to pregnant women getting the COVID-19 vaccine, what would be some reasons why they rejected?
18. What scares people about pregnant women getting the COVID-19 vaccine?
19. What are some of the beliefs about the COVID-19 vaccine during pregnancy that you have heard in your community?
20. Where do people in your community get their information around the COVID-19 vaccine from?
21. Thank you for your time. Is there anything else you would like to share about the COVID-19 vaccine in pregnancy, or COVID-19 during pregnancy in general?

**Interview guide – Supervisors: Nurse supervisors and other sub-county health managers**

1. Can you tell me a little about yourself and your role here at the health facility or health system (county or sub-county)? How long have you been working at this health facility? In your current role?
2. What have you heard about COVID-19 in pregnancy? About the COVID-19 vaccine for pregnant women?
3. Where did the nurses and other staff you supervise learn about COVID-19 in pregnancy?
4. Where did the nurses and other staff you supervise learn about the COVID-19 vaccine for pregnant women? Did they receive any special training?
5. When did your facility (or region) start giving COVID-19 vaccines (month-year)? As an estimate, how many COVID-19 vaccines have been provided to pregnant women at this health facility or region?
6. If there was a pregnant woman at an ANC in this facility (or facility in the region) who was interested in getting a COVID-19 vaccine, please tell me what would happen - walk me through the process or the various steps.
7. In your opinion, what delays pregnant women from getting a COVID-19 vaccine?
8. In your opinion, what helps pregnant women to get a COVID-19 vaccine?
9. From what you hear from the nurses and other staff providing the COVID-19 vaccine, how frequently do pregnant women accept the COVID-19 vaccine? For example, if there were five pregnant women, how many do you think would accept the COVID-19 vaccine?
10. Has acceptance for COVID-19 vaccine during pregnancy changed between when it was first rolled out till now? Why or why not?
11. 11. From what you hear from the nurses providing the COVID-19 vaccine, what are some of the reasons why pregnant women accept the COVID-19 vaccine? Any examples?
12. From what you hear from the nurses providing the COVID-19 vaccine, what are some of the reasons why pregnant women refuse the COVID-19 vaccine? Any examples?
13. What beliefs about the COVID-19 vaccine during pregnant can be found in the community? Are there any beliefs that enable pregnant women to get the COVID-19 vaccine? Any beliefs or misconceptions that reduce COVID-19 vaccine uptake among pregnant women?
14. Where are pregnant women and their families getting information about the COVID-19 vaccine during pregnancy from? Where are people hearing misconceptions from?
15. Are there disagreements between health workers about pregnant women getting the COVID-19 vaccine? Can you share any examples?
16. Thank you for your time. Is there anything else you would like to share about the COVID-19 vaccine in pregnancy, or COVID-19 during pregnancy in general?
