# Supplementary material for: “Now that the baby is out, I can be vaccinated”: a qualitative study on COVID-19 vaccine hesitancy in pregnant women in Kilifi, Kenya
Source: Front Public Health. 2026 Feb 6;14:1730282. doi: 10.3389/fpubh.2026.1730282 (PMC12920558; doi:10.3389/fpubh.2026.1730282)
Supplement: Supplementary file 2 [file Table_1.DOCX]

**“Now that the baby is out, I can be vaccinated”: A qualitative study on COVID-19 vaccine hesitancy in pregnant women in Kilifi, Kenya.**

**Supplementary material S2 - Working group on Vaccine Hesitancy Determinants Matrix**(1)

1. MacDonald NE. Vaccine hesitancy: Definition, scope and determinants. Vaccine. 2015 Aug;33(34):4161–4.

| **Contextual influences**  **Influences arising due to historic, socio-cultural, environmental, health system/institutional, economic, or political factors** | Communication and media environment |
| --- | --- |
|  | Influential leaders, immunization programme gatekeepers |
|  | Historical influences |
|  | Religion / culture / gender / socio-economic |
|  | Politics / policies |
|  | Geographic barriers |
|  | Perception of the pharmaceutical industry |
| **Individual and group influences**  **Influences arising from personal perception of the vaccine or influences of the social/peer environment** | Personal, family and/or community members’ experiences with vaccination, including pain |
|  | Beliefs, attitudes about health and prevention |
|  | Knowledge / awareness |
|  | Health system and providers – trust and personal experience |
|  | Risk/benefit (perceived, heuristic) |
|  | Immunization as a social norm vs not needed / harmful |
| **Vaccine / vaccination – specific issues directly related to vaccine or vaccination** | Risk/benefit (epidemiological and scientific evidence) |
|  | Introduction of a new vaccine or new formulation or a new recommendation for an existing vaccine |
|  | Mode of administration |
|  | Design of a vaccination programme / Mode of delivery (e.g., routine programme or mass vaccination campaign) |
|  | Reliability and/or source pf supply of vaccine and/or vaccination equipment |
|  | Vaccination schedule |
|  | Costs |
|  | The strength of the recommendation and/or knowledge base and/or attitude of healthcare professionals |
